# Supplementary material for: Host factors, inflammatory markers, and clinical outcomes of Naegleria fowleri meningoencephalitis
Source: Commun Med (Lond). 2026 May 19;6:290. doi: 10.1038/s43856-026-01658-8 (PMC13186982; doi:10.1038/s43856-026-01658-8)
Supplement: Supplementary file 2 — Supplementary Information [file 43856_2026_1658_MOESM2_ESM.docx]

**Supplementary Materials**

*Host factors, inflammatory markers, and clinical outcomes of Naegleria fowleri meningoencephalitis*

# Supplementary Figure S1: Patient flow diagram

**↓**

Suspected meningoencephalitis cases identified through enhanced surveillance [n = 247]

**↓**

Laboratory testing performed (CSF microscopy + real-time qPCR for N. fowleri 18S rRNA) [n = 247]

**↓**

Not confirmed: Negative microscopy and PCR (n=39); Alternative diagnosis established (n=8) [n = 47]

**↓**

Laboratory-confirmed PAM cases enrolled: PCR positive only (n=103, 51.5%); Microscopy and PCR positive (n=97, 48.5%) [n = 200]

**↓**

Resolved outcomes: Deaths (n=61); Recoveries (n=73) [n = 134 (67.0%)]

**↓**

Under treatment at database closure [n = 66 (33.0%)]
